# Epidemiology of invasive meningococcal disease, Japan, 2013 to 2023

**DOI:** 10.2807/1560-7917.ES.2024.29.46.2400136

**Published:** 2024-11-14

**Authors:** Miho Kobayashi, Hajime Kamiya, Munehisa Fukusumi, Hideyuki Takahashi, Yukihiro Akeda, Motoi Suzuki, Tomimasa Sunagawa

**Affiliations:** 1 Center for Field Epidemic Intelligence, Research and Professional Development, National Institute of Infectious Diseases, Tokyo, Japan; 2 Gunma Prefectural Institute of Public Health and Environmental Sciences, Gunma, Japan; 3 Center for Surveillance, Immunization, and Epidemiologic Research, National Institute of Infectious Diseases, Tokyo, Japan; 4 Department of Bacteriology I, National Institute of Infectious Diseases, Tokyo, Japan

**Keywords:** *Neisseria meningitidis*, meningococcal disease, surveillance, epidemiology, Japan

## Abstract

**Background:**

The National Surveillance for Invasive Meningococcal Disease (IMD) initiative started in Japan in April 2013. Multiple international mass gathering events have since been held in Japan, and the COVID-19 pandemic has occurred.

**Aim:**

We summarised 10 years of national surveillance data for IMD in Japan to describe epidemiological characteristics of IMD and evaluate the influence of mass gatherings and the COVID-19 pandemic on IMD.

**Methods:**

Upon diagnosis of IMD, patient information and specimens were collected and reported to local health centres. We analysed the epidemiology of IMD cases reported between 1 April 2013 and 31 March 2023.

**Results:**

Among 274 cases reported (median age: 55 years; 55% male), no outbreaks related to mass gathering events were identified. The annual reported incidence of IMD was 0.001–0.039 cases per 100,000 individuals between 2014 and 2022, with a notable decrease after 2020. The overall case fatality rate was 12% (33/274). The most frequent serogroups were Y and B (46 and 17%). Multilocus sequence typing revealed a predominance of clonal complex (cc) 23, followed by cc2057, while cc11 was detected in eight cases.

**Conclusion:**

The reported incidence of IMD in Japan is low compared with high-endemic countries and decreased further during the COVID-19 pandemic. This unique epidemiology of IMD in Japan lacks a clear explanation. However, distribution of meningococcal strains, such as predominance of serogroup Y, could be a contributing factor. Maintaining high-quality surveillance, including of serogroups and sequence types, is crucial to manage and prevent future IMD cases in Japan effectively.

**Key public health message**

**What did you want to address in this study?**

We wanted to describe the epidemiology of invasive meningococcal disease (IMD) in Japan and to evaluate the influence of mass gathering events and the COVID-19 pandemic on the frequency of this disease. The study looked at surveillance data collected over 10 years (April 2013 to March 2023).

**What have we learnt from this study?**

Japan reported 274 IMD cases in this period, with the numbers declining from 2020 onwards. The number of IMD per 100,000 individuals was low (0.001–0.039) during 2014 to 2022. The case fatality rate was 12%; no deaths occurred among individuals aged 14 years or younger. Importantly, no cases of IMD were associated with mass gathering events, indicating that such events did not contribute to an increase in IMD incidences in Japan.

**What are the implications of your findings for public health?**

The incidence of IMD in Japan has been low and has further decreased since the onset of the COVID-19 pandemic. However, with the easing of COVID-19 restrictions and changes in peoples’ behaviour, there is a potential for an increase in IMD cases and the occurrence of outbreaks. Careful monitoring of the trends of causal pathogens is necessary to effectively control IMD outbreaks.

## Introduction

Invasive meningococcal disease (IMD) caused by *Neisseria meningitidis* is transmitted through respiratory droplets [1]. The incidence of IMD varies by country and region, with the highest rates observed in sub-Saharan Africa [2]. While IMD can affect individuals of all ages, it is more common in children and adolescents, and its symptoms progress rapidly [3]. Outbreaks of IMD are often reported in crowded environments, such as student dormitories and mass gathering events [4,5].

*Neisseria meningitidis* is classified into 12 serogroups [6], with most invasive disease cases caused by serogroups A, B, C, W, X and Y [2,6]. In Japan, the meningococcal conjugate vaccine (MCV4) targeting serogroups A, C, W and Y was first approved in 2015, and two MCV4s (Menactra and MenQuadfi) are currently available for vaccination [7]. However, these vaccines are not included in the routine vaccination programme in Japan, and no vaccine for serogroup B has been approved [7]. *Neisseria meningitidis* is commonly classified as sequence types (STs) by multilocus sequence typing (MLST) that assigns unique allelic profiles to seven loci for STs, which are further grouped into clonal complexes (ccs) [8]. Symptoms and disease severity may vary depending on the specific ST, such as ST-11 cc11, associated with hyper-invasiveness [9].

In Japan, the national surveillance for meningococcal meningitis was initiated in 1999 [10]. In 2013, the reporting criteria were revised to include surveillance for IMD, including bacteraemia and other invasive diseases [10]. In 2014, the annual incidence of IMD was reported to be 0.028 cases per 100,000 population [11]. Since then, there have been mass gatherings in Japan, including international events such as the Rugby World Cup and the Tokyo 2020 Olympic and Paralympic Games. In addition, there have been behavioural changes, such as physical distancing, entry and exit restrictions to Japan and the implementation of personal precautions in the years of the COVID-19 pandemic [12]. However, the impact of these events on IMD incidence remains unclear.

This study aimed to summarise the national surveillance data for IMD in Japan, spanning from April 2013 to March 2023. Our objectives were to describe the epidemiological characteristics of IMD and assess the impact of mass gatherings and the COVID-19 pandemic on its incidence.

## Methods

### Data collection

In Japan, national surveillance for meningococcal meningitis was initiated in 1999 under Japan’s Infectious Diseases Control Law [10]. Since April 2013, not only meningitis but also bacteraemia caused by *N. meningitidis* have been reported as IMD [10]. To be notifiable as a case of meningococcal meningitis, the patient must have symptoms compatible with meningitis, such as headache, vomiting, impaired consciousness, neck stiffness and bulging anterior fontanelle, and *N. meningitidis* must be detected in samples from normally sterile sites. However, as of the 2013 revision of the reporting criteria, only blood and cerebrospinal fluid (CSF) have been considered eligible specimens for this purpose. 

In 2015, there was an outbreak of IMD caused by serogroup W (MenW) linked to a mass gathering event, specifically the World Scout Jamboree that took place in Japan [4]. Several participants from abroad developed IMD. No Japanese participants developed IMD, but one female Japanese who was on the flight to Europe with symptomatic participants developed a high fever and arthritis, and meningococci with the same sequence type as the cases were detected from her joint fluid. However, it went unreported at the time because only cases in whom meningococci were detected in blood or spinal fluid met the criteria for reporting [4,10]. 

To improve the quality of surveillance, the reporting criteria for IMD were expanded in November 2016 to include other sterile sites such as joint and pericardial fluid, in addition to blood and spinal fluid [10]. Physicians diagnosing IMD are required to report the demographic information, symptoms, outcomes, vaccination history and exposure history of a case to the public health centres with jurisdiction where the hospital is located. Since 2015, the patient's name, address and contact information have also been required in the report; the public health centres could conduct active case finding, contact tracing and health monitoring of contacts to prevent the spread of the disease. Data on cases are entered into the National Epidemiological Surveillance of Infectious Diseases (NESID) system by the public health centres, compiled by each prefecture, and finally aggregated by the National Institute of Infectious Diseases (NIID).

This study analysed and summarised the reported IMD data collected between 1 April 2013 and 31 March 2023. We conducted inquiries with local jurisdiction to ensure the completeness of the data, including outcomes, vaccination history and exposure history. In addition, we also asked the local infectious disease surveillance centres for information on the spread of the disease, including the number of close contacts.

The annual reported incidence per 100,000 individuals was determined using the estimated population on 1 October for each year [13]. In addition, we analysed the average annual reported incidence per 100,000 individuals by prefecture.

### Analyses of *Neisseria meningitidis* isolates, serogrouping and MLST

In Japanese infectious diseases surveillance, the sample from an IMD case is usually sent to the local public health institute nearest to the location of the medical facility, for serogroup testing. The samples are then sent to the NIID for MLST. However, it is important to note that reporting serogroups in the NESID system is not mandatory. Therefore, some local public health institutes do not perform serogroup testing due to the lack of technical staff with expertise. In such instances, the NIID proactively contacts the local public health institutes to share the samples from these cases and conducts both serogrouping and MLST.

*Neisseria meningitidis* isolates were incubated on GC agar plates for 18 h at 37 °C with 5% CO_2_. Serogrouping was performed using either PCR [14] or the slide agglutination method with anti-meningococcal rabbit serum (DIFCO or RAMEL). While the latex agglutination test (Pastorex Meningitis assay kit, Bio-Rad Laboratories) was also used in hospital laboratories, it is essential to note that this method could not distinguish between serogroups Y and W. Furthermore, to determine the ST, MLST was performed at the NIID, following previously described protocols [8].

## Results

### Demographic characteristics

The NESID system included 274 reported IMD cases during the study period (Table 1). Among the patients, 55% (150/274) were male, and the median age was 55 years (interquartile range: 32–71 years). Meningitis accounted for approximately two-thirds of reported IMD cases (167/274; 61%). Although 14 cases were reported among individuals residing in dormitories or social welfare facilities, none exhibited secondary infections or outbreaks.

**Table 1:** Demographic characteristics of reported invasive meningococcal disease cases, Japan, 1 April 2013–31 March 2023 (n = 274)

Characteristics		n	%
Sex			
Male		150	55
Female		124	45
Age (years)			
Median (Interquartile range)		55 (32–71)	
Clinical circumstances			
Death^a^		33	12
Meningitis^b^		167	61
Meningitis^b^	Detection in CSF	31	11
	Detection in CSF and blood	36	13
	Detection in blood	100	36
Bacteraemia		101	37
Others^c^		6	2
Cases’ situation			
Living in a dormitory, shared accommodation, or older adult care facility		14	5
Imported case^d^		6	2
Vaccination			
Yes		4	1
No		66	24
Not reported		204	74
Serogroup			
Y		126	46
B		47	17
C		16	6
W		11	4
Y or W		6	2
Not groupable		7	3
Unknown/not tested		61	22

^a^ Based on the information at the time of reporting.

^b^ Includes cases of concomitant bacteraemia.

^c^ Two cases of arthritis, one case of disseminated intravascular coagulation and multiple organ failure, one case of fever and cough, one case of abdominal pain, one case of fever.

^d^ Those who had travelled overseas within 10 days of onset.

Data as of 5 April 2023.

Vaccination history was collected for 70 (26%) cases; among them, only four cases had received the vaccine against meningococcal disease. One case had a record of being vaccinated with Menactra (MCV4), but the vaccine type for the other three was unknown. However, only MCV4 is currently approved and available in Japan; therefore, we assumed that they had also received MCV4.

**Figure 1:**
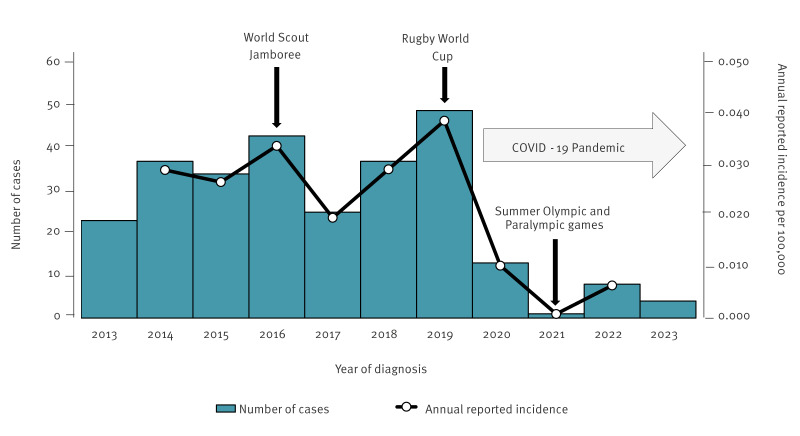
Number of reported invasive meningococcal disease cases, Japan, 1 April 2013–31 March 2023 (n = 274)

Between 2014 and 2019, 25–49 cases were reported annually, while only 13, one and eight cases were reported in 2020, 2021 and 2022, respectively (Figure 1). The annual reported incidence between 2014 and 2022 was 0.001–0.039 per 100,000 individuals. Overall, no specific area of high incidence was observed, but there was a trend towards higher rates in populated urban areas.

Data as of 5 April 2023. In 2013, the number of reports was for 9 months and in 2023, the number of reports was for 3 months.

### Impact of mass gathering events

Between 2013 and 2023, several international mass gathering events were held in Japan. Among these events, one case of IMD was reportedly linked to the Rugby World Cup in 2019 [15]. The patient arrived in Japan from Australia and developed IMD 16 days after arrival. The IMD strain identified in the patient's blood and CSF was serogroup B, ST-213. Until then, only one case of ST-213 had been reported in Japan in 2018. The identified ST-213 corresponded to the prevalent type in Australia [16]; therefore, this patient was likely to have introduced the strain from their country of origin. Notably, no secondary cases were observed during that period.

During the Tokyo 2020 Olympic and Paralympic Games in 2021, no IMD cases were reported. Before the event, additional measures were undertaken to proactively prevent IMD. These included conducting a risk assessment, vaccinating the participants and volunteers with MCV4, as well as implementing enhanced surveillance to mitigate the potential for infectious disease outbreaks [17,18]. Furthermore, although the games were held without spectators to prevent the spread of COVID-19, the playbook established the principle of minimising physical interactions and wearing masks [18].

### Distribution of age and case fatality rate

**Figure 2:**
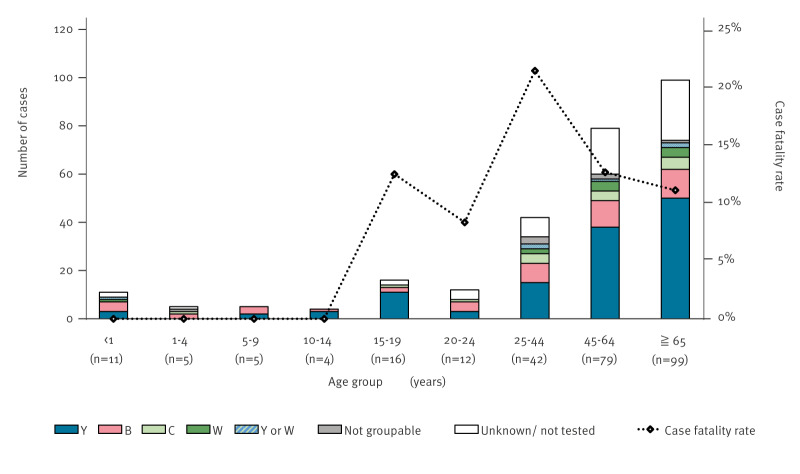
Distribution by serogroup, age group, and case fatality rate of reported invasive meningococcal disease cases, Japan, 1 April 2013–31 March 2023 (n = 273^a^)

^a^ Excluding one case of unknown age.

Most reported cases (248/273; 91%) were aged 15 years or older. The overall case fatality rate (CFR) was 12% (33/274), with no fatal cases reported since 2020. The highest CFR (21%) occurred in the age group 25–44 years, while no deaths were reported in those under 14 years (Figure 2). Overall, the proportion of MenY was the highest (46%). However, the serogroup distribution among different age groups, particularly for individuals under 24 years of age, remained unclear due to the limited number of cases in these specific age groups.

Data as of 5 April 2023. Outcome is based on information at the time of reporting.

### Serogroups and sequence types

Regarding the serogroups of IMD cases diagnosed between April 2013 and December 2018, 64% (99/155) were serogroup Y (MenY) and 15% (23/155) were serogroup B (MenB). In contrast, between January 2019 and March 2023, 47% (27/58) were classified as MenY and 41% (24/58) as MenB. The distribution of age groups was similar between the two time periods.

Results from MLST were available for 56% (153/274) of cases (Table 2). The cc23 was found to be the dominant cc, followed by cc2057. Eight cases were identified as cc11, and four of the eight were classified as serogroup C; these were reported in 2013, 2014, 2018 and 2022. The remaining cc11 cases were classified as serogroup W and were reported between 2017 and 2019. Notably, one IMD case with serogroup W cc11 had travelled to Europe 2 days before the onset of symptoms. The other cc11 patients had no history of travel abroad or contact with patients confirmed to have IMD.

**Table 2:** Clonal complexes and serogroups identified, Japan, 1 April 2013–31 March 2023 (n = 153)

Clonal complex	Serogroups	n	%
cc23	Y	93	61
cc2057	B	17	11
cc41/44	B	10	7
cc11	W	4	3
	C	4	3
cc213	B	5	3
cc32	B	1	1
	Non-groupable	4	3
cc4821	B	1	1
	C	1	1
cc167	Y	1	1
cc178	Non-groupable	1	1
Others		11	7

Data as of 5 April 2023.

## Discussion

We summarised the national surveillance data for IMD for 10 years to understand its epidemiology in Japan, including assessment of the impact of multiple international mass gatherings and the COVID-19 pandemic on IMD in the country.

National surveillance for IMD in Japan was launched in April 2013. At that time, the annual incidence of the disease was lower than that in other high-income countries such as United States, European countries and Australia , with a rate of 0.028 per 100,000 individuals in 2014 [11]. Since 2016, the definition of reportable cases has been expanded to include instances where *N. meningitidis* has been detected in samples from normally sterile sites other than blood or spinal fluid [10]. Despite this change, our results showed that the number of reported IMD cases in Japan remained low, ranging from ca 20 to ca 40 cases per year (corresponding to a reported incidence rate of 0.001–0.039 per 100,000 population). This is less than other invasive bacterial infections; 1.078–2.650 per 100,000 population of invasive pneumococcal disease and 0.155–0.430 per 100,000 population of invasive *Haemophilus influenzae* disease were reported annually in the same period [19]. The CFRs for IMD in our study were comparable with those reported in other countries. The CFR was the highest among individuals aged 25–44 years in Japan, contrary to the expected pattern of a higher CFR in older adults [20]. In addition, no deaths were reported among individuals under 14 years, a unique characteristic of IMD in Japan as reported previously [11]. Thus, the reported incidence in Japan was low, and the CFR was similar to that in other countries; however, the age group with a high CFR was different from that in foreign countries.

Our results indicate that the incidence of IMD in Japan was consistently low throughout the study period, although the exact reason is unclear. This result differs from previous reports from other countries, where outbreaks and increased incidence of IMD have been observed in connection with mass gatherings [2,21]. Although ca 1.7 million spectators attended the Rugby World Cup, only one case of IMD was reported during the event [15]. In 2021, 420,000 people attended the Tokyo Olympics and 310,000 attended the Tokyo Paralympics. We assume that there have not been any IMD cases associated with the events worldwide, as we have received any International Health Regulations (IHR) report on IMD from other countries at this point [22]. While the Olympic and Paralympic Games took place without spectators, some Olympic officials and athletes did contract COVID-19 [18], which raises the possibility that there could also have been cases of IMD, which, like COVID-19, is transmitted by droplet infection. Notably, there were no IMD cases at these events, although the number of attendees was much higher than at the World Scout Jamboree which did have reported IMD cases. 

It is suggested that risk factors for IMD are associated with behaviours such as alcohol consumption and smoking [2,23,24] as well as complement deficiencies, including C5–9 [25]. In particular, C9 deficiency is found in 0.1% of the Japanese population [25] and may have the potential to influence IMD. However, it is not required to report these lifestyle behaviours and underlying conditions for national surveillance. Therefore, we could not evaluate the causal relationship between these risk factors and the low incidence of IMD at this point. However, the low incidence rate of IMD in Japan may be explained by the existence of a universal health insurance system, which lowers the hurdle to visiting a medical institution and makes it easier to receive appropriate treatment in a timely manner. Nevertheless, even if these hypotheses are correct, the fact that only two outbreaks of IMD in Japan have been detected since surveillance began [4,11] indicates that differences in the nature of the bacteria may have more influence than the patients’ background and behaviours.

The global distribution of serogroups exhibits geographical variation, with MenB a major causative agent in certain regions such as North and South America, Australia, and Europe [2]. These areas frequently report outbreaks of MenB in universities and other high-risk environments [5]. MenY is the dominant serogroup in Japan; however, the proportion of MenB cases in recent years has increased. The underlying causes of this shift remain unclear. The coverage rate for the meningococcal conjugate vaccine in countries where it is included in the routine vaccination schedule is relatively high (e.g. 70–80% for the United Kingdom [26]), but in Japan, the coverage rate is estimated to be notably low because it is not listed as a routine vaccine [7]. Thus, it is unlikely that vaccine selection pressure plays a significant role. The possibility of an increase in IMD cases due to MenB and potential outbreaks in the future cannot be ruled out, and the approval of the MenB vaccine in Japan should be seriously considered at the earliest.

Our MLST analysis revealed that MenY cc23 was dominant, followed by cc2057. MenY cc23 previously increased in the United States and Europe but is no longer dominant [27], and MenB cc2057 may be unique to Japan [28]. In contrast, cc11, which is known for its hyperinvasive potential [9], was detected in only eight IMD cases. In addition, two MenW cc11 cases related to an international mass gathering event were identified in 2015 [4]; however, these cases were not reported to the NESID system because of the surveillance reporting criteria at the time. Given its association with large outbreaks in other regions, such as the Hajj [29], it is unclear why the reported incidence of cc11 IMD cases in Japan is low and sporadic. The cc41/44 and cc32 clonal complexes, also recognised as hyperinvasive lineages [9], have been infrequently reported in Japan. The unique distribution of meningococcal strains in Japan may influence the epidemiology of IMD. Continuous monitoring of serogroups and MLST profiling is crucial for detecting changes in the epidemiology of IMD and for early detection of outbreaks.

The COVID-19 pandemic has been associated with a substantial decrease in reported cases of invasive bacterial diseases, including IMD, and Japan was not an exception [30,31]. Preventive measures such as physical distancing, mask-wearing and travel restrictions in Japan during the pandemic may have contributed to reducing the spread of meningococcal infections and imported cases. As such, IMD cases related to international mass gatherings did not occur during the COVID-19 pandemic. However, entry and exit restrictions to Japan to prevent the spread of COVID-19 were abolished in October 2022. Moreover, from May 2023, the government no longer requested or involved infection control based on the law [12]. Currently, infection control measures, including wearing masks, are left to the voluntary efforts of the public [12]. In other countries that deregulated COVID-19 measures earlier than Japan, invasive bacterial infections, including IMD, are rising. For example, the number of IMD cases in England has increased since the end of 2022, reaching the same level as before the pandemic [32]. If we observe an increased incidence of IMD after the relaxation of the COVID-19 measures, we could assume that the low incidence before the pandemic may have been further reduced by the COVID-19 preventive measures in Japan; however, the post-pandemic development need to be analysed in future studies. Therefore, close and continuous monitoring of the epidemiology of IMD in Japan is important.

This study has four limitations. Firstly, physicians may not always list IMD as a distinct diagnosis, particularly due to the limited number of domestic IMD cases. This practice could potentially result in under-detection. However, once meningitis or sepsis is suspected by a physician, access to laboratory tests such as PCR and culture is high, at a reasonable cost. Given the rapid progression of IMD and its severity, a substantial number of cases are less likely to remain undetected. Secondly, fatal IMD cases could be under-reported if an individual was alive at the time of physician diagnosis and subsequently died. However, we actively inquired with local jurisdiction as we complied the data to ensure that no fatalities were missed. Therefore, the reported CFR for IMD is less likely to be underestimated. Thirdly, not all isolates were analysed; however, as 78% (213/274) of the reported cases underwent serogroup testing and 72% (153/213) had MLST results, the results were more complete for the reported cases than in a previous report [11]. Finally, we performed only MLST, whole genome sequence data were not available. Further examination, including resistance profiles, would be required in the future.

## Conclusions

A clear explanation for the low reported incidence of IMD and the specific patient epidemiology in Japan remains unknown. The reported incidence of IMD decreased during the COVID-19 epidemic, and there was no outbreak of IMD related to mass gathering events. The unique distribution of meningococcal strains in Japan may be a contributing factor. Similar to other infectious diseases, the transmission of meningococci may increase as countermeasures for COVID-19 are relaxed. Moreover, the low vaccination rate for IMD in Japan represents a potential risk of outbreaks. Therefore, it is crucial to maintain high-quality surveillance and closely monitor the serogroups and ccs of patients with IMD to control and prevent severe cases and outbreaks effectively.
